# Microarrays Highlight Tumor–Connective Tissue Interactions in Cancer Outcomes

**DOI:** 10.1371/journal.pbio.0030220

**Published:** 2005-05-10

Of the 200 or so diseases collectively known as cancer, tumors of the soft tissue include some of the rarest and most diverse. Malignant soft tissue tumors, or sarcomas, and benign soft tissue tumors—which together comprise over 100 subtypes—take root in immature nerve and mesenchymal cells, which give rise to muscle, fat, cartilage, and other connective tissues. Reflecting the ubiquitous nature of connective tissue, soft tissue sarcomas can occur nearly anywhere in the body. Pathologists classify soft tumor sarcomas based on the proteins they express and on their resemblance to normal connective tissue cells (such as adipocytes, smooth muscle cells, and fibroblasts): tumors consisting of cells with cytologic features of fat cells, for example, are called liposarcomas while those forming the spindle-shaped, organized sheets typical of smooth muscle cells are called leiomyosarcomas.

But many connective cells appear too similar and express too many of the same proteins for traditional screens to distinguish among them. Assays are further complicated by the admixture of non-malignant cells, including inflammatory cells and those recruited to form new blood vessels, into the soft tissue tumor landscape.

In a new study, Robert West, Matt van de Rijn, and their colleagues investigate the notion that different types of fibroblastic tumors mirror the features of normal fibroblasts and search for tissue markers that might distinguish them. Using DNA microarrays, the authors profiled gene expression patterns in two types of fibroblastic tumors and found significant differences in the expression of functionally related genes, confirming that each tumor carries a unique genetic signature. These gene sets also appear in the matrix of normal connective tissue, or stroma, leading to the identification of two noncancerous fibroblast subtypes.

The tumors analyzed in this study—solitary fibrous tumor (SFT) and desmoid-type fibromatosis (DTF)—behave differently but consist of cells that look similar under the microscope, making them well suited to the task of identifying novel connective tissue markers. Typically benign, SFT tumors respond well to surgical excision and are thought to arise either from fibroblasts, most frequently in the thoracic cavity. DTFs, aggressive tumors found deep within the soft tissue of the trunk, abdomen, or extremities, are difficult to excise completely and are also thought to arise from fibroblasts.

Gene arrays taken from ten DTF tumors and 13 SFT tumors showed that each tumor type had distinct gene expression patterns reflecting different gene functions. For example, DTF gene profiles included many genes involved in fibrosis (scarring) and extracellular matrix remodeling, a prerequisite for the invasive behavior of aggressive DTF tumors. SFTs, on the other hand, express many genes involved in synthesis and maintenance of the basement membrane that surrounds muscle cells, blood vessels, and other specialized cells. Based on these variable expression patterns, the authors hypothesized that the tumors' cells of origin might perform different functions in normal tissue.[Fig pbio-0030220-g001]

**Figure pbio-0030220-g001:**
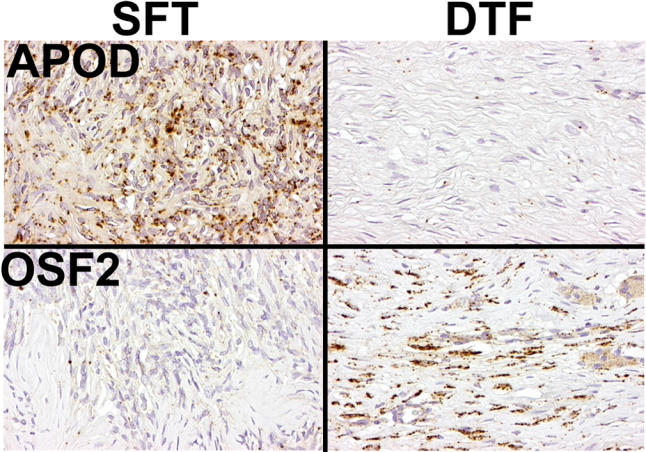
Tissue samples at 600× magnification show that these two soft tissue tumors express different protein markers: solitary fibrous tumors express the APOD protein marker (top), and desmoid-type fibromatosis tumors express OSF2

Next, West et al. examined gene expression patterns in fibroblasts found in normal tissue samples to look for tumor-specific markers. As predicted, DTF markers were found in one set of tissues—related to scarring and inflammation—and SFT markers were found in another—breast and skin fibroblasts, and benign breast growths. The authors' use of soft tissue tumors to define different subsets of stromal cells is similar to past studies that used lymphomas to discover novel subsets of normal lymphoid cells.

Because normal fibroblasts can contribute to cancer progression—by providing the cellular matrix, or stroma, that supports tumor growth—the authors looked for DTF- or SFT-specific markers in the stroma of breast cancer patients. Two groups, the authors note, showed differences in the expression of DTF and SFT genes. Patients with tumors showing high DTF gene expression had the best prognosis, suggesting that tumor–stromal interactions might affect disease progression.

The difficulty of classifying fibroblasts has complicated efforts to understand how fibroblasts aid tumor progression. This study suggests that breast cancers with different stromal signatures may have different clinical outcomes, which raises many questions for future study. If the same tumor type grows on a different stromal background, will it progress differently? If so, how do tumor–stromal interactions influence progression? Thanks to the fibroblast markers identified here, scientists have new tools for exploring these questions.

